# The epileptologist's perspective of focal cortical dysplasia type 3: From concept to management

**DOI:** 10.1111/epi.70023

**Published:** 2025-11-24

**Authors:** André Palmini, Francine Oliveira, Eliseu Paglioli, Harvey Sarnat, Ricardo Paganin, Ricardo Soder, William Martins, Rafael Paglioli, Thomas Frigeri, Fernanda Schuh, Vicenzo Zarpellon

**Affiliations:** ^1^ Porto Alegre Epilepsy Surgery Program Neurology and Neurosurgery Services, Hospital São Lucas, Pontifícia Universidade Católica do Rio Grande do Sul (PUCRS) Porto Alegre Brazil; ^2^ Department of Clinical Neurosciences, School of Medicine Pontifícia Universidade Católica do Rio Grande do Sul (PUCRS) Porto Alegre Brazil; ^3^ Brain Institute (InsCer) PUCRS Porto Alegre Brazil; ^4^ Department of Pathology, Faculty of Medicine Universidade Federal do Rio Grande do Sul Porto Alegre Brazil; ^5^ Department of Paediatrics (Neurology) Alberta Children's Hospital Research Institute (Owerko Centre), University of Calgary Cumming School of Medicine Calgary Canada; ^6^ Department of Pathology and Laboratory Medicine (Neuropathology) Alberta Children's Hospital Research Institute (Owerko Centre), University of Calgary Cumming School of Medicine Calgary Canada; ^7^ Department of Clinical Neurosciences Alberta Children's Hospital Research Institute (Owerko Centre), University of Calgary Cumming School of Medicine Calgary Canada

**Keywords:** classification, clinical relevance, epileptologist, FCD type 3, focal cortical dysplasia

## Abstract

The recent International League Against Epilepsy (ILAE) official and updated classification of focal cortical dysplasia (FCD) includes a third type—FCD type 3—characterized by architectural abnormalities (cortical dyslamination) associated with another “principal” lesion: hippocampal sclerosis (HS), developmental tumors, vascular malformations, or gliotic scars. We posit that the clinical relevance of FCD type 3 is not established, as dyslamination cannot be reliably identified preoperatively and (although unproven, because unidentifiable) persistence of cortical tissue with putative dyslamination after resective surgery does not preclude seizure control. Here we discuss these issues from the epileptologist's perspective and stimulate the debate on whether the FCD type 3 construct impacts decision‐making in the epilepsy surgery field—or else is of little practical significance.

**Key points**

- Useful classification schemes should have clinical applications for treatment strategies, prognosis, or both.
- Focal cortical dysplasia (FCD) type 3 is of necessity a heterogeneous category because subtypes have distinct pathogeneses and associated principal lesions have distinct epileptogenic potentials.
- Cortical dyslamination cannot be identified in vivo, even through high‐resolution magnetic resonance imaging; thus, the role of putative dyslaminated cortex on epileptogenesis is unclear.
- Because presurgical identification is lacking, current surgical strategies for patients with refractory seizures and the principal lesions presumably associated with FCD type 3 do not take into account the possibility of cortical dyslamination.
- The clinical relevance of the FCD type 3 category is currently unclear, and further developments in imaging andgenetics are still needed to prove or disprove the validity of the type 3 construct.

## 1 FROM HIPPOCAMPAL SCLEROSIS TO FOCAL CORTICAL DYSPLASIA AND ITS CLASSIFICATIONS

In the 1980s and early 1990s, the establishment of many epilepsy surgery centers around the world was driven by the delineation of the electroclinical features of temporal lobe epilepsy (TLE) and the subsequent in vivo identification of hippocampal sclerosis (HS) on magnetic resonance imaging (MRI).^1^, ^2^, ^3^ At that time, resective epilepsy surgery was essentially TLE surgery.^4^

However, pari passu with advancements in MRI, malformations of cortical development (MCDs) and, particularly, focal cortical dysplasia (FCD), slowly gained traction as a relevant cause of refractory epilepsy. By the turn of the century, HS and FCD rivaled in prevalence in surgical series and, currently, FCD is recognized as the main neocortical pathology underlying refractory focal seizures in both children and adults.^5^, ^6^

The prominence gained by FCD brought several parallel developments, such as the realization that refractory focal neocortical epilepsy with normal MRI is frequently a synonym of FCD under the microscope, leading to efforts to advance MRI techniques that could unveil small, albeit highly epileptogenic, dysplastic lesions.^7^, ^8^, ^9^, ^10^, ^11^ Moreover, since FCD was established as the key neuropathology behind neocortical refractory focal epilepsy, attention has been heightened toward deviations from normal cortical architecture as possible subtypes of FCD. However, because the “classic” FCD lesions have grossly abnormal cellular elements (Taylor‐type^12^ or cytoarchitectural dysplasia from the Milan group nomenclature^13^) the accommodation of purely architectural cortical abnormalities under the FCD umbrella demanded a classification to avoid mixing disorders with largely distinct histology (as well as distinct electroclinical, imaging, and molecular genetic features, as later became clear). Several suggestions in that direction were made in the late 1990s and early 2000s, and the proposal from a panel of epileptologists and neuropathologists leading to what came to be known as the “Palmini classification” (classifications are often named after the first author of the original publication) eventually prevailed.^14^

The so‐called Palmini classification acknowledged that FCD lesions could have grossly abnormal cellular elements or not, thus proposing two types of FCD: type 1, with only architectural abnormalities, and type 2, with dysplastic neurons accompanied or not by balloon cells. Despite its simplicity, that classification was well received and paved the way to electro‐clinical‐imaging‐molecular genetic correlations. Although still in development, quite a lot of progress have been made based on the “type 1 vs type 2” dichotomy.^15^, ^16^

Heightened attention to cortical architectural abnormalities as the underlying pathology responsible for refractory seizures led to two refinements that are the focus of this commentary: one was the appreciation that cortical dyslamination could have more than one configuration that, theoretically, could relate to specific epileptological scenarios. The other was bringing under the FCD umbrella observations that cortical architecture occasionally deviated from the well‐organized six‐layered pattern when associated with other pathologies (themselves causes of refractory epilepsies).

These two refinements from the Palmini classification were the pillars of a new classification for FCD—with the official support of the International League Against Epilepsy (ILAE).^17^ In this new classification, FCD type 1 incorporates three configurations of dyslamination: vertical microcolumns (1A), tangential (1B), or mixed (1C). Moreover, a third FCD category was created: when a principal epileptogenic lesion is associated with cortical architectural abnormality (dyslamination), the patient is said to have FCD type 3. Thus, dyslamination associated with HS, tumors, vascular malformations, or scars from hypoxic ischemic insults correspond to FCD subtypes 3A, 3B, 3C, and 3D, respectively. In other words, in 2011, the ILAE specialist panel decided to put dyslamination associated with other lesions on the spot.^17^ As one of us wrote in an editorial, future developments would show whether such focus on that context of dyslamination would pay off in a clinically relevant way, as the simpler type 1 vs type 2 dichotomy did.^18^

Of interest, when the ILAE classification was recently updated, it turned out that what needed to be updated was the incorporation of two entities unrelated to cortical dyslamination: (i) a formal delineation of mild malformations of cortical development –(or mMCDs), in relation to the quantification of heterotopic neurons on the white matter, and (ii) the description and clinical relevance of mMCDs with oligodendroglial hyperplasia and epilepsy (MOGHE).^19^, ^20^ In other words, very little progress has been seen with the “dyslaminations,” particularly when associated with principal lesions, that is, FCD type 3.

Because pragmatic decisions must be made on how to evaluate and operate on the many patients with refractory seizures harboring those principal lesions (HS, tumors, vascular malformations. or scars), which may or may not be associated with adjacent dyslamination, we believe it useful to provide an epileptologist's perspective of FCD type 3. We start with revisiting some conceptual issues and then address the main clinical challenges that must be faced to decide whether FCD type 3 should effectively be considered an entity with clinical relevance, thus impacting patient management.

## 2 CONCEPTUAL ISSUES REGARDING FCD TYPE 3

### 2.1 Pooling distinct mechanisms of developmental and acquired dyslamination into a single entity and the clinical meaning of the cortical dyslamination associated with a principal lesion

There has been tremendous progress in the understanding of the fascinating neurobiological orchestration that leads to the formation of the human neocortex.^21^ A large array of genes controls the timing and extent of several overlapping stages, from neuroblast and glial proliferation in the periventricular germinative zone to their intimate relationship during outward migration and subsequent neuroglial detachment, allowing migrating neurons to occupy precise cortical positions. During this journey, neurons establish cortico‐cortical and subcortical synaptic connections that eventually lead to the architectural organization of the mature neocortex.^22^, ^23^ This delicate sequence of events can be interfered with by genetic abnormalities or environmental insults, and the resulting abnormality will be a malformation of cortical development (or MCD). Timing, type, and extent of the interference are crucial variables for the pathogenesis of the resulting malformation.^24^

It is generally accepted that in patients with cortical malformations restricted to the layering of the neocortex, without accompanying gross cellular abnormalities, initial stages of neuroglial differentiation, proliferation, and outward migration have passed uneventfully, and that the resulting dyslamination occurred later in the process of corticogenesis.^24^ However, in contrast to cortical dysplasia without associated principal lesions (i.e., FCD types 1 and 2), which represent truly developmental abnormalities of the cortex, dyslamination associated with other lesions may represent secondary phenomena driven by the principal lesion—and thus challenging the connection with the other two types of FCD.^25^ More importantly, should this be so, the epileptogenic potential of such cortex with dyslamination becomes unclear. In other words, if these architectural abnormalities occur after the neocortex was already formed, caused by the deleterious effects of another lesion—the backbone of FCD type 3—then the sheer concept of dysplasia comes into question. This conceptual conundrum may impact presurgical and surgical strategies and therefore deserves attention.

It should be clear that FCD type 3 is a pure histopathological category, in which lesions of diverse nature and pathogeneses have the associated dyslamination (although itself histologically distinct) as the sole common denominator.

One can foresee at least two, non‐mutually exclusive, pathogenetic scenarios for the dyslamination associated with principal lesions:

- Cortical dyslamination occurring during development in conjunction with, but likely *not caused* by, a principal lesion.

It is possible that this is the mechanism of the dyslamination associated with HS or tumors, particularly developmental tumors. In patients with TLE and HS, Thom and colleagues pointed to striking neuronal loss in cortical layers II and III of the lateral temporal neocortex.^26^ One can hypothesize that in this scenario, dyslamination is acquired or reorganizational, probably caused by the same underlying insult or vulnerability that leads to HS—but *not occurring because of the HS*. Similarly, some types of developmental tumors, notably dysembryoplastic neuroepithelial tumor (DNET) and ganglioglioma, to name the more prevalent lesions, are associated with dysplastic cortical abnormalities at variable distance from the tumor. We have discussed this before, raising the issue of whether such association represents two distinct pathologies (i.e., dual pathology), or a single pathology with a gradient of abnormalities, in which parts of the lesion do not have tumoral cells but only dysplastic abnormalities.^27^ We also discussed then—and will expand those considerations below—whether dyslamination associated with tumors (or HS) have epileptogenic relevance and how could it be identified or suspected preoperatively, as that would impact upon management.

ii Cortical dyslamination occurring in close relation to the principal lesion during or after cortical development (post‐migratory) and likely driven by the lesion.

**FIGURE 1:**
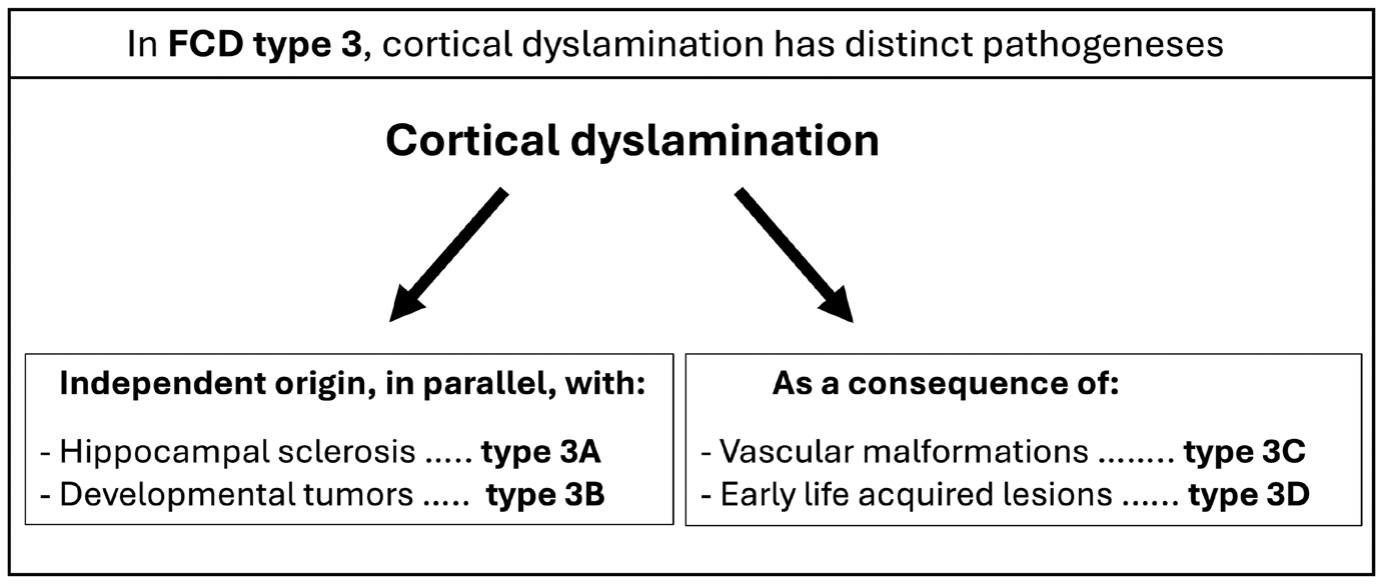
Schematic representation of putative pathogenetic mechanisms underlying dyslamination in the various subtypes of focal cortical dysplasia (FCD) type 3.

It is probable that dyslamination in the context of vascular malformations, including the leptomeningeal angiomatosis of Sturge–Weber syndrome and that associated with hypoxic–ischemic insults, represent cortical reorganization following primary insults by these lesions. These are essentially acquired dyslaminations, due to direct injury to one or more cortical layers, as suggested by high‐resolution histopathological studies.^28^ It is possible that mechanisms of plasticity play a role in the re‐accommodation of neuronal and glial cells following injury, and the only reason for the abnormal cortical architecture thus resulting to be called “dysplasia” is that these events occur early (either during cortical formation late in gestation or early in the neonatal period) and contribute to the reshaped anatomy of the brain. However, as discussed later, it is impossible to determine by imaging the extent of cortex within or adjacent to these gross lesions that lacks normal lamination. In fact, one should perhaps assume that these lesions and surrounding cortex do have abnormal cortical layering as a natural consequence of early injury—and, if, so, the terminology FCD type 3C or 3D is just another way to name the abnormal cortex related to these lesions (and NOT independent malformations in themselves). The fact that this abnormal, scarred cortex generates seizures is hardly surprising and has been known since Penfield's era, and presurgical or intra‐operatory recordings must identify such relevant epileptogenic tissue.^29^ Figure 1 schematically displays these two pathogenetic scenarios.

In summary, it is inescapable to understand FCD type 3 as a heterogeneous group of entities, including some in which the defining dyslamination seems to represent a true cortical malformation, such as adjacent to developmental tumors; others in which although architectural abnormalities emerge during development they likely represent distinct parts of an underlying insult (a reasonable hypothesis for the dyslamination associated with HS^26^); and others in which dyslamination is secondary to the pathogenesis of the principal lesion (post anoxic–ischemic and vascular malformations). It would thus be surprising if these disparate entities had common epileptological grounds. As suggested in the recent text of the updated ILAE classification,^19^ time will tell whether a significant gap between histopathology and clinical practice in FCD type 3 will remain, particularly regarding the doubtful dysplastic nature and epileptogenic relevance of dyslamination associated with heterogeneous principal lesions.

## 3 CAN FCD TYPE 3 BE IDENTIFIED BEFORE SURGERY? REVIEWING THE YIELD OF MRI

A quick answer to the question is that identification of the cortical dyslamination by imaging is often very difficult or impossible, as we examine below.

### 3.1 Looking into dyslamination associated with HS and developmental tumors

A good example of the difficulty to identify cortical dyslamination by imaging is the inconsistent correlation between dyslamination and even high‐field (7T) MRI in the study by Garbelli et al^30^ They correlated ex vivo imaging of temporal neocortex with microscopic findings in specimens resected from 13 patients with TLE and HS, 9 of whom also had dyslamination, that is, FCD type 3A. In beautiful images, they showed that normal cortical microarchitecture displayed both a striking line of hypointensity in layer IV and distinct signal intensities in supragranular compared to infragranular layers. One or both features were expected to be absent in samples with dyslamination, yet some patients with dyslamination had normal imaging features and others with abnormal imaging features had normal histopathology. In other words, even 7T MRI could not reliably identify dyslamination in temporal neocortex associated with HS.

Because of the potential impact upon presurgical evaluation and extent of resection in patients with temporal lobe epilepsy and refractory seizures, the quest for imaging identification of dyslamination in FCD type 3A did not stop there. We and others hopefully hypothesized that the abnormal signal in the white matter of the anterior temporal lobe seen in some patients with HS could represent disorganized fiber tracts related to neocortical dyslamination.^31^, ^32^ In our recently published series, increased white matter signal represented vacuolization and increased water content amid myelinated fibers in the anterior temporal lobe; yet cortical lamination did not differ in patients with and without such white matter abnormality.^32^ Similar findings were reported a decade earlier in an elegant electron microscopy study by Garbelli and colleagues.^31^ Thus, distinct studies reached the same conclusion that although the abnormal white matter signal did represent myelin abnormalities, the overlying cortex was well laminated and thus such imaging finding does not help identification of dyslamination (i.e., FCD type 3A).

A more optimistic perspective regarding imaging identification of dyslamination could be had when analyzing FCD type 3B, in which dyslamination is associated with a tumor. Optimism emerges from the well‐known imaging finding of blurred cortical–subcortical transition adjacent to developmental tumors, particularly DNET.^33^, ^34^, ^35^ In fact, dyslamination in this context has raised the issue of whether it should be seen as a single lesion with distinct histological abnormalities in different lesion compartments (akin to a histopathological gradient) or if dyslaminated cortex and tumor are two separate lesions, that is, dual pathology.^27^ The concept of FCD type 3B does not solve this conundrum because FCD refers only to the dyslaminated cortex, and the ”type 3” is simply because a tumor is nearby. This is made even clearer by recent findings of FCD‐like architectural cortical abnormalities adjacent to diffuse gliomas^36^: cortical dyslamination was “unexpectedly” found adjacent to 25% of gliomas whose excision provided enough tissue to examination, and there was no hint of this cortical architectural abnormality on imaging. Furthermore, and even more unexpectedly, the authors reported similar dyslamination in the cortical tissue of 40% of patients in a control group with no neoplasms and no history of epilepsy—and no imaging abnormality as well.

Therefore, the initial optimism regarding the identification of the dyslamination in patients with FCD type 3B should be tempered by these latter findings that, in fact, suggest that dyslamination adjacent to tumors may be non‐epileptogenic. Furthermore, the abnormal imaging suggestive of FCD adjacent to DNETs is perhaps a confirmation of the well‐known fact that these developmental tumors are part of a “dysplastic continuum.”^33^, ^34^

### 3.2 The lesion complex of FCD types 3C/3D: What imaging shows and what dyslamination represents

**FIGURE 2:**
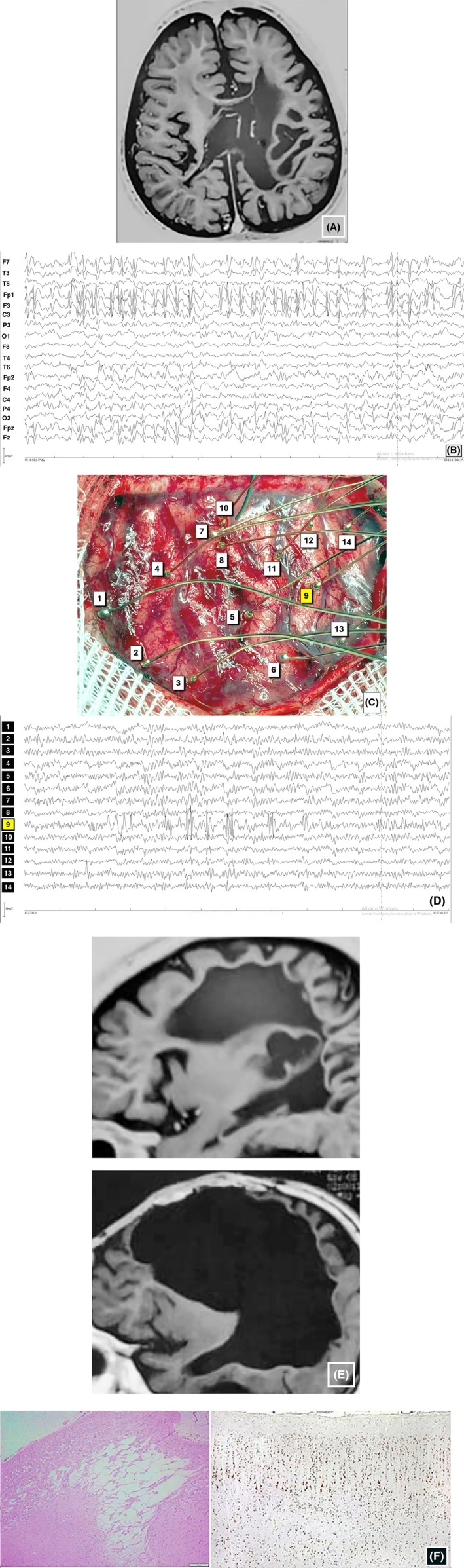
A 9‐year‐old boy with severe focal motor seizures frequently evolving into bilateral tonic‐clonic attacks and recurrent bouts of epilepsia partialis continua. He was born after 26 weeks of gestation weighing 900 g, had periventricular hemorrhage, needed 4 months of neonatal intensive care unit care, and evolved with right hemiparesis and significant psychomotor delay. (A) Axial inversion recovery magnetic resonance imaging (MRI) section showing bilateral encephaloclastic lesions, worse on the left. (B) Scalp electroencephalography (EEG). Despite bilateral MRI lesions, there was strong predominance of epileptiform abnormalities in the left frontal region. (C) Intraoperative cortical view of the left fronto‐central regions. (The left frontal pole is to the right of the reader). Numbers correspond to electrocorticography(ECoG) ballpoint electrodes. An ECoG sample is shown in D, pointing to maximal abnormality at electrode Position 9, in the left frontal region. (E) Shows pre‐ and post‐resection sagittal T1‐weighted MRI sections. Note the extensive left frontal lobe resection. (F) H&E and NeuN (x10) histological sections showing the destructive lesion and normal lamination of adjacent cortex. The boy has been seizure‐free for 4 years, since operation.

The issue of identifying cortical dyslamination associated with a principal lesion is made even more difficult in individuals with FCD types 3C and 3D. Vascular malformations and hypoxic–ischemic insults damage the cortex in a centrifugal fashion, with a core lesion at the center and epileptogenic scar tissue at the borders. MRI details this “lesion complex” and does not specifically identify cortex with abnormal lamination. Therefore, whatever cortical architectural abnormalities are found in this context should be seen as resulting from the underlying pathogenesis of the lesion complex, that is, secondary to vascular or hypoxic–ischemic insults. Furthermore, from the standpoint of presurgical evaluation and delineation of surgical strategies, the most relevant epileptogenic cortical tissue contained in the lesion complex must be identified by whatever method and resected, irrespective of whether parts of it lacks normal lamination (Figure 2).

## 4 DISTINCT EPILEPTOGENIC RELEVANCE OF DYSLAMINATION IN FCD TYPE 1 AND FCD TYPE 3

It is important to consider a likely distinct epileptogenic relevance of dyslamination according to whether an associated (principal) lesion is present, or the architectural abnormalities are the sole lesion. Excessive neurons in vertically arranged microcolumns may impact intracortical hyperexcitability, leading to seizures, particularly in children with FCD type 1A.^37^ Furthermore, different centers have reported patients with refractory seizures who became seizure‐free following cortical resection, and in whom cortical dyslamination (i.e., FCD type 1) was the only underlying histopathology.^38^ Thus, the question *is not* whether isolated dyslamination can be epileptogenic, but rather whether dyslamination associated with an unequivocal main epileptogenic lesion has clinical relevance.

Furthermore, it is widely recognized that these patients with isolated FCD type 1 have the poorest outcomes in resective epilepsy surgery. For instance, in the series of Tassi et al.^38^, an Engel class I outcome was reported in 46% of patients with isolated FCD 1 (i.e., dyslamination) vs 82% when dyslamination was associated with a principal lesion (tumor or HS, currently classified as FCD type 3). Thus, seizure‐free rates in patients later shown to have dyslamination in whom the principal lesion was resected (i.e., FCD type 3) are much better than those with isolated FCD type 1.^38^ Similarly, in a large series of more than 350 patients with FCD by Jayalakshmi and colleagues,^39^ 90% of patients with dyslamination plus a principal lesion (i.e., FCD type 3) were seizure‐free, compared to only 50% of those with isolated FCD type 1. Because the main reason suggested for poorer outcomes following surgery of patients with FCD type 1 is the purportedly more diffuse distribution of dyslamination, should the same apply to cases with dyslamination in which a principal lesion was present, results should be similarly poor. However, exactly the opposite happens. This discrepancy strongly suggests that dyslamination associated with a principal lesion is not as relevant.

It is important to realize that the relations between concepts and pragmatic clinical issues in medicine are complex, particularly when the direction of the vector is from concept to patient (and not the other way round). Pertaining to the discussion on the distinct epileptogenic role of dyslamination in FCD types 1 and 3, sophisticated data on DNA methylation of surgical specimens suggested specific patterns for all subtypes of FCD, including FCD type 3.^40^ However, DNA methylation is an ubiquitous biological phenomena dependent on multiple factors, and it is not surprising that distinct patterns would be found in FCD type 3 associated with various principal lesions, which both produce and impinge upon the dyslaminated cortex in specific ways. Therefore, the fact that DNA methylation can diagnose FCD subtypes and have distinct profiles for the FCD type 3 subtypes does not translate into the epileptogenicity of the associated dyslamination.

## 5 RELATION BETWEEN SURGICAL RESULTS AND PERSISTENCE OF CORTEX WITH PUTATIVE DYSLAMINATION

For FCD types 3C and 3D, (but also to some extent 3A and 3B), there would be practical advantages should (i) cortex with dyslamination be identified by imaging and (ii) consistent evidence indicate that such dyslamination is epileptogenic. Epileptologists are well versed on the difficulties of making decisions on the extent of resection when facing patients with refractory seizures due to the lesion complexes associated with Sturge–Weber, other vascular malformations, and gliotic anoxic–ischemic scars. Very often the surrounding abnormal cortex encroaches upon eloquent or indispensable areas, and should architectural epileptogenic abnormalities be identified beforehand, surgical planning would be simplified. However, as discussed above, MRI shows the lesion to be complex, with a mixture of atrophic gyri and white matter destruction (Figure 2). Whether dyslamination will be found under the microscope is intrinsic to parts of the lesion complex, and its contribution to the epileptogenicity is not known before resection. That being said, parts of the gliotic tissue surrounding the vascular abnormalities in FCD type 3C and the encephaloclastic lesions in FCD type 3D are epileptogenic in themselves and must be identified through neurophysiological methods, irrespective of whether dyslamination is present.

A pragmatic way to investigate the epileptogenicity of putative cortical dyslamination associated with principal lesions is to review the outcome regarding seizure control in patients with refractory seizures in whom resection left behind parts of the would‐be abnormally laminated cortex. Inferences on the epileptogenicity of the latter could then be made from such results. Below we make the point that the epileptogenicity of dyslamination associated with principal lesions should be questioned by reviewing patient series and providing clinical examples showing that preservation of putatively dyslaminated cortex does not preclude seizure control. Furthermore, we advance that whatever neurophysiological abnormalities are recorded by whatever means of evaluation (scalp electroencephalography [EEG], stereo‐EEG [SEEG], or electrocorticography [ECoG]) is likely related to the principal lesion and its impact upon adjacent tissue—and has little to do with a truly, independent dysplastic abnormality. Specifically we show that patients may be seizure‐free despite persistence of cortex with putative dyslamination in four clinical scenarios, one for each subtype of FCD type 3—moving from A to D.

### 5.1 FCD type 3A

Should dyslamination in the temporal neocortex be consistently epileptogenic in patients with HS, one could imagine that in those who become seizure‐free after temporal lobe surgery despite preservation of the temporal neocortex microarchitecture would probably be normal, that is, no dyslamination and no FCD type 3A. However, that theoretical concoction does not match epidemiological findings. A well‐studied, unselected series of patients who had anterior temporal lobectomy (ATL) for TLE associated with HS showed that 25% had neocortex with dyslamination, that is, FCD type 3A.^41^ Assuming this percentage, it is difficult to explain that in a very large series of more than 600 patients with TLE and HS who had either ATL or selective amygdalohippocampectomy (SAH), the latter had only slightly reduced rates of seizure freedom, despite persistence of the temporal neocortex.^42^ Bringing this issue home, we found the same rates of seizure freedom in two studies, one comparing results with the two techniques in 161 patients operated by the same neurosurgeon,^43^ and the other extending the outcome into a survival analysis spanning 18 years.^44^ Irrespective of the technique (selective or nonselective), the latter analysis showed that 77% of patients were classified as Engel outcome class I.^44^

This issue of surgical results with selective vs nonselective temporal lobe surgery is certainly relevant to the debate on the epileptogenicity of dyslaminated temporal neocortex, that is, FCD type 3A, because many still believe that SAH has an inferior outcome compared to ATL. Therefore, it is worth examining two meta‐analyses which support the view that dyslamination in the temporal neocortex does not usually have clinical relevance. Jain and colleagues^45^ performed a network metanalysis including 19 series, and in 12 (63%) SAH had similar or better results than ATL. In another review, Hu et al.^46^ included 10 of the 19 series reviewed by Jain and colleagues, and in 4, SAH was superior, in 3, both techniques had similar rates of success, and in only 3 was ATL was superior.^46^ In this latter detailed meta‐analysis, there were only seven publications considered of high quality (6 to 8 stars in the Newcastle–Ottawa Scale quality score): four favored selective approaches, two ATL, and one showed similar results with both techniques. From a neurophysiological standpoint, Fauser and Schulze‐Bonhage showed that in patients with HS and neocortical dyslamination most seizures recorded by intracranial electrodes did start in the mesial structures—which challenges the epileptogenic impact of dyslamination in most patients.^47^ Therefore, although the jury is still out, there is sufficient controversy to suggest that epileptogenicity of temporal neocortex associated with HS is often not clinically significant.

The validity of the FCD type 3 category is further challenged by findings of minimal if any impact of associated dyslamination in the clinical presentation or seizure outcome of patients operated for refractory epilepsy due to a “principal lesion.” For instance, Fauser et al. reported that patients with FCD types 1 and 3A presented with similar clinical features.^48^ Nearly 10 years later, Cossu et al. showed in a large multicentric series of 220 patients with HS that no differences emerged in the anatomo‐electro‐clinical profile and surgical results between patients with FCD type 3A and with isolated HS.^49^ Thus, they pointed to the limited clinical relevance of FCD type 3A in HS‐related epilepsy. Finally, the fact that clinical features did not differ in patients with FCD type 1 in the temporal lobe and those with FCD type 3A suggests that the latter category adds little for the clinical epileptologist, particularly because imaging cannot identify the neocortical dyslamination.

### 5.2 FCD type 3B

Some studies suggest that gross total resection of developmental tumors suffice to control seizures, without the need for additional resection of adjacent neocortex. This has been shown in gangliogliomas^4^ ^,^ ^50^ and in some types of DNETs, as described in detail by Chassoux and colleagues.^33^, ^34^ Regarding the latter, surgical strategy does not take into account the possibility of dyslamination, but rather the presumed extent of tumoral and dysplastic cells. In the most comprehensive series of DNETs to date, 93% patients were in Engel outcome class I following complete removal of the tumor. From the FCD type 3 perspective, it is unlikely that dyslamination would not be present in at least some of these patients, and in those scenarios would not have any bearing upon outcome, suggesting a low degree of epileptogenicity. Although the authors found that completeness of resection of the epileptogenic zone was also related to surgical outcome, resection of the principal lesion was the key factor in most cases^33^ – and cortical dysplasia was usually mixed with the tumor in the “dysplastic DNET types.”

### 5.3 FCD type 3C

**FIGURE 3:**
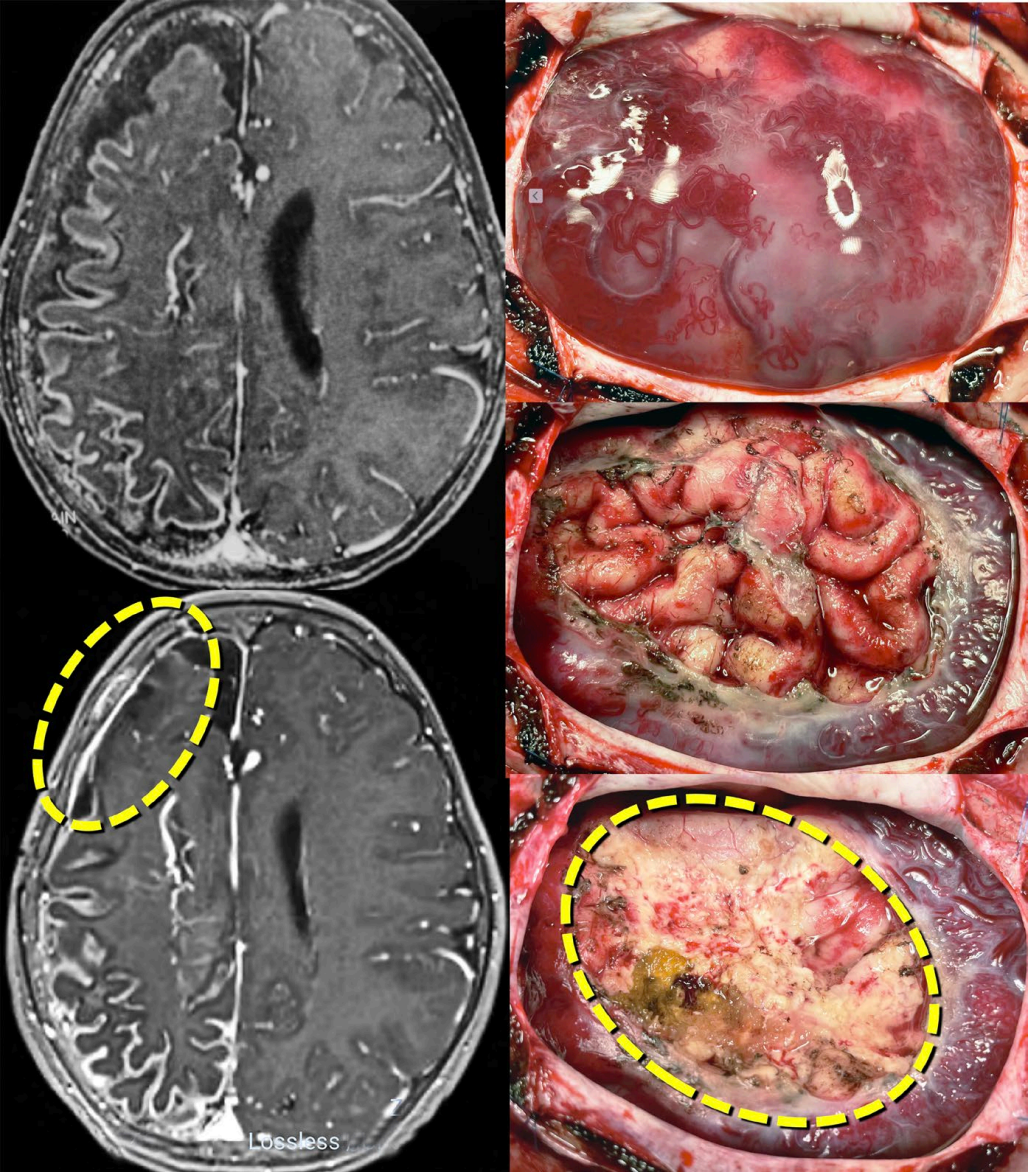
Composite picture of gadolinium‐enhanced T1‐weighted axial magnetic resonance imaging sections and intraoperative views of a 7‐year‐old girl with recurrent seizures associated with right hemisphere pial angiomatosis characteristic of Sturge–Weber syndrome. Despite the extensive lesion, motor function in the left hemibody was only mildly affected and the girl was fully functional. Maximal EEG discharges involved the right frontal lobe, which was significantly atrophied. A focal resection was made, as shown in the dashed circles both in the image and the exposed brain—leaving most of the angiomatosis in place. She has been seizure‐free for the last 3 years, since surgery, and playing accordion.

There is some debate on the best surgical strategy for patients—usually children—with Sturge–Weber disease and refractory seizures, in whom the pial angiomatosis is extensive in one hemisphere and encroaches upon the primary sensorimotor cortex. Despite some contralateral weakness, many patients retain function in the paretic hand and walk unassisted. The question then arrives as to whether the preferred approach would be an hemispherectomy, disconnecting the whole damaged cortex (that could have associated dyslamination—FCD type 3C) or a partial resection, removing part of the lesion complex, but preserving areas that involve functional cortex. There is no generalizable answer from patient series,^51^, ^52^ although case reports do suggest that seizure freedom can be achieved with partial resections of the lesion.^53^ We have operated on two patients who are seizure‐free for 2 or more years, despite partial resection (Figure 3).

### 5.4 FCD type 3D

**FIGURE 4:**
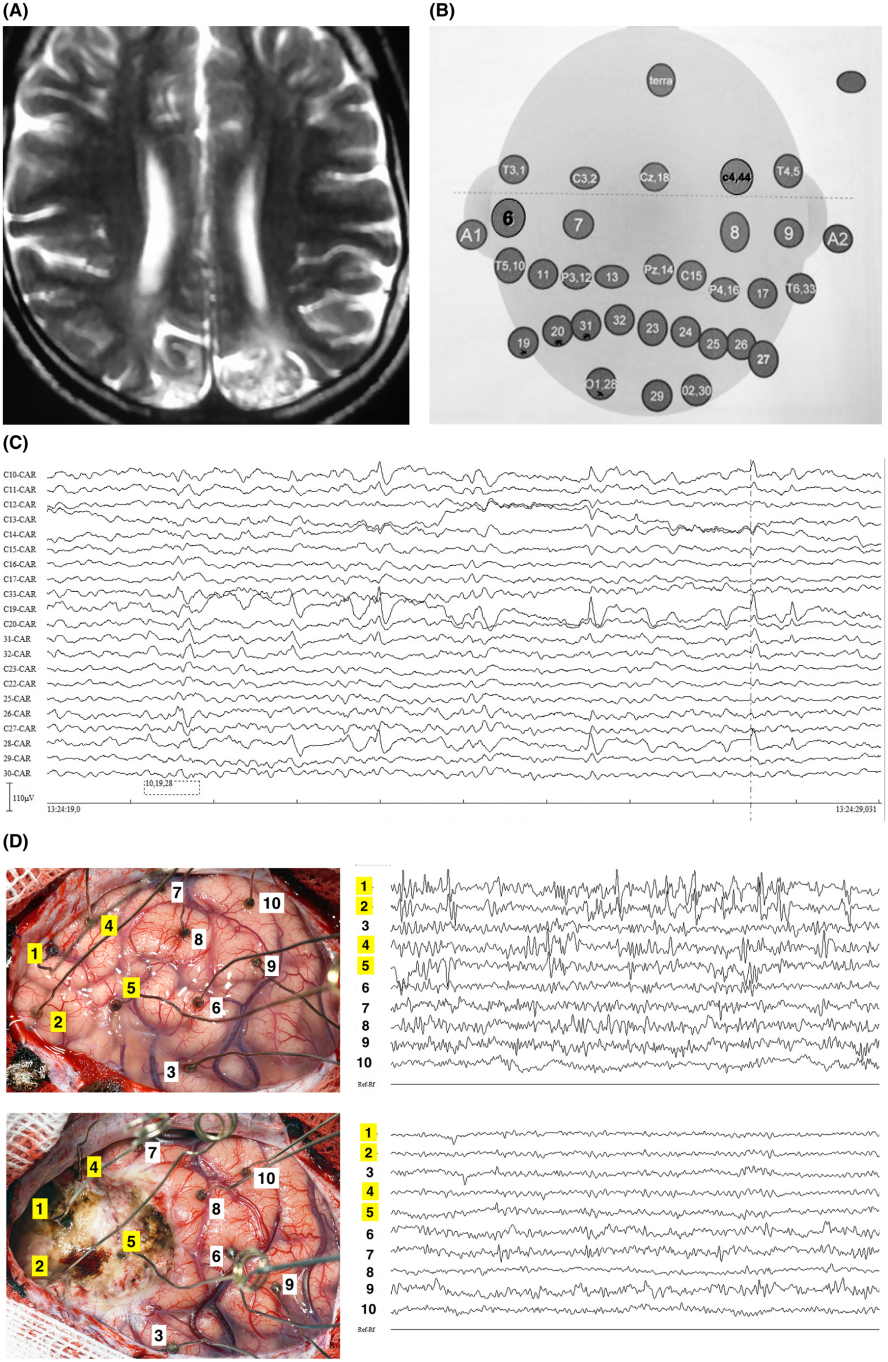
A 26‐year‐old man with a history of neonatal sepsis and seizures since infancy. When evaluated, he had medically refractory focal impaired consciousness seizures and occasional evolution into bilateral tonic‐clonic attacks. (4A) 1.5T axial T2‐weighted magnetic resonance (MR) image showing bilateral gliotic lesions in the parieto‐occipital lobes, larger on the left. (4B) An approximate map of very closely spaced electrodes focusing on the posterior head regions. Electrode numbers on the scalp EEG epoch in monopolar montage shown in 4C refer to this map. Note the highest amplitude of epileptiform discharges focally at electrode positions 19 and 28, at the left temporo‐occipital region. That was a consistent finding throughout the evaluation, with seizures recorded from the same region. (4D) Composite picture of intraoperative cortical view and electrocorticographic (ECoG) recording pre‐ and post‐ resection. In the upper row, pre‐resection ECoG shows frequent discharges in the left occipito‐temporal region (electrode Positions 1, 2, 4, and 5). In the lower row, note the disappearance of discharges following resection.

Several studies report patients such as this man we have been following for more than 15 years, with bilateral gliotic parieto‐occipital lesions secondary to early diffuse insults, who became seizure‐free following resection of one gliotic lesion, yet leaving the contralateral lesion in place (Figure 4). Should dyslamination be a frequent finding adjacent to these gliotic scars (FCD type 3D), it would be reasonable to anticipate a similar abnormality in the contralateral, non‐resected lesion produced by the very same pathogenesis, the persistence of which did not preclude complete seizure control in this man and many other patients with similar bilateral ulegyria who had unilateral resection.^54^, ^55^

## 6 PRACTICAL ISSUES OF MANAGEMENT

### 6.1 Presurgical / pre‐resection evaluation and surgical strategies

Classifications have many uses, and in the context of the histopathology of a neurological disorder such as FCD the main usefulness of a classification is to create a base of knowledge combining clinical, electrographic, imaging, and genetic scenarios with the resulting outcome of interventions. Once such base of knowledge is created, it may then become possible to anticipate the most likely results of a given medical or surgical intervention.

Because imaging has a very low yield in identifying dyslamination, and the clinical electrographic scenarios of FCD type 3 do not differ from those of the principal lesion (with or without adjacent dyslamination), it has not been possible to establish a clinical relevance for the FCD type 3 construct—with the sole exception of the previously well‐known situation that some DNETs are indeed associated with adjacent dysplasia, and identification of the latter may be possible by imaging.^33^, ^34^

Furthermore, the retrospective identification of the dyslamination—and the establishment of a post‐surgical FCD type 3 diagnosis—does not appear to bear upon surgical outcome. Thus, from a practical standpoint, the clinical relevance of FCD type 3, that is, what really matters for the treating physicians, will have to await both consistent preoperative diagnosis and the demonstration that such knowledge will impact strategies to delineate the most relevant epileptogenic tissue and extent of resection. Should that eventually be possible, patients with tumors, vascular abnormalities, and gliotic scars will benefit, as reliable preoperative histopathological anticipation may help decide on the extent of resection. Until then, the identification and resection of the relevant epileptogenic tissue will continue to be accomplished by clinical, imaging, and neurophysiological methods independent of the FCD type 3 construct.

## 7 FINAL CONSIDERATIONS

Perhaps a good way to close this commentary is by acknowledging that the construct of FCD type 3 has expanded the very concept of dysplasia, incorporating acquired disorganization of the cortex associated with or secondary to a principal epileptogenic lesion.^56^ However, unlike FCD types 1 and 2, FCD type 3 should be seen as heterogeneous from many perspectives. The associated principal lesions are heterogeneous and have distinct pathogeneses, leading to distinct patterns of dyslamination. Moreover, the presurgical suspicion of the dyslamination by MRI is seldom possible. Finally, many years into the establishment of the ILAE classification and an update elaborated by a panel of experts, the degree of epileptogenicity (if any) of the cortical dyslamination associated with the principal lesions is still unknown.

When the Palmini classification was proposed, it was already clear that patients with FCD lesions harboring dysmorphic neurons with or without accompanying balloon cells did have a distinct clinical, electrographic, and imaging profile.^57^, ^58^ Thus, FCD type 2 was already born with unequivocal clinical relevance, and FCD type 1 was proposed as a part of the dysplastic spectrum that would wait for similar profiling from further developments in clinical imaging correlations (as later proved to be the case in a specific subgroup of children^37^). In contrast, FCD type 3 is a histopathological undertaking, without clinical, imaging, or neurophysiological hallmarks. Therefore, it is hardly surprising that the unspecific findings in these patients cannot inform on therapeutic strategies.

Essentially, one could conceive of FCD type 3 as an entity prone to misdiagnosis, because it waits for consistent imaging features of the dyslaminated cortex, neurophysiological markers of epileptogenicity, specific genetic abnormalities (despite some advancements in methylation abnormalities^59^) and, perhaps more importantly, to achieve a level of clinical relevance that will help decision‐making in epilepsy surgery—such as has been the case with FCD type 1A, another type of dyslamination, unaccompanied by principal lesions.^37^ Meanwhile, the FCD type 3 construct should be seen as an observation of the impact of largely distinct lesions upon cortical layering at different stages of cortical development.

## AUTHOR CONTRIBUTIONS

A.P. conceptualized the paper and wrote the first draft. E.P., R.P., T.F., F.H., R.P., R.S., W.M., V.Z., and F.M. reviewed the manuscript and proposed substantial changes on histopathological, imaging, surgical, and neurophysiological perspectives that led to the final form to be submitted.

## FUNDING INFORMATION

Research productivity scholarship # 314492/2023‐2 – PQ 1A to AP.

## CONFLICT OF INTEREST STATEMENT

A.P. has received honoraria for speaking and consultancy from, Abbott, Adium, Aché, Apsen, Eurofarma, GreenCare, EaseLabs, FQM, Libbs, Novartis, Prati‐Donaduzzi, and UCB. W.M. has received honoraria for speaking Adium, Libbs, Torrent, and UCB. F.H., E.P., R.P., T.F., F.M., R.S., R.P., and V.Z. have nothing to disclose.

## ETHICAL PUBLICATION STATEMENT

We confirm that we have read the Journal's position on issues involved in ethical publication and affirm that this report is consistent with those guidelines.

## Data Availability

This is a critical review paper including the mention of clinical evolution and imaging findings of selected patients. Data on these patients are available from the authors upon request.
